# Supplementary figures and images for: Multivalency, autoinhibition, and protein disorder in the regulation of interactions of dynein intermediate chain with dynactin and the nuclear distribution protein
Source: eLife. 2022 Nov 23;11:e80217. doi: 10.7554/eLife.80217 (PMC9771362; doi:10.7554/eLife.80217)

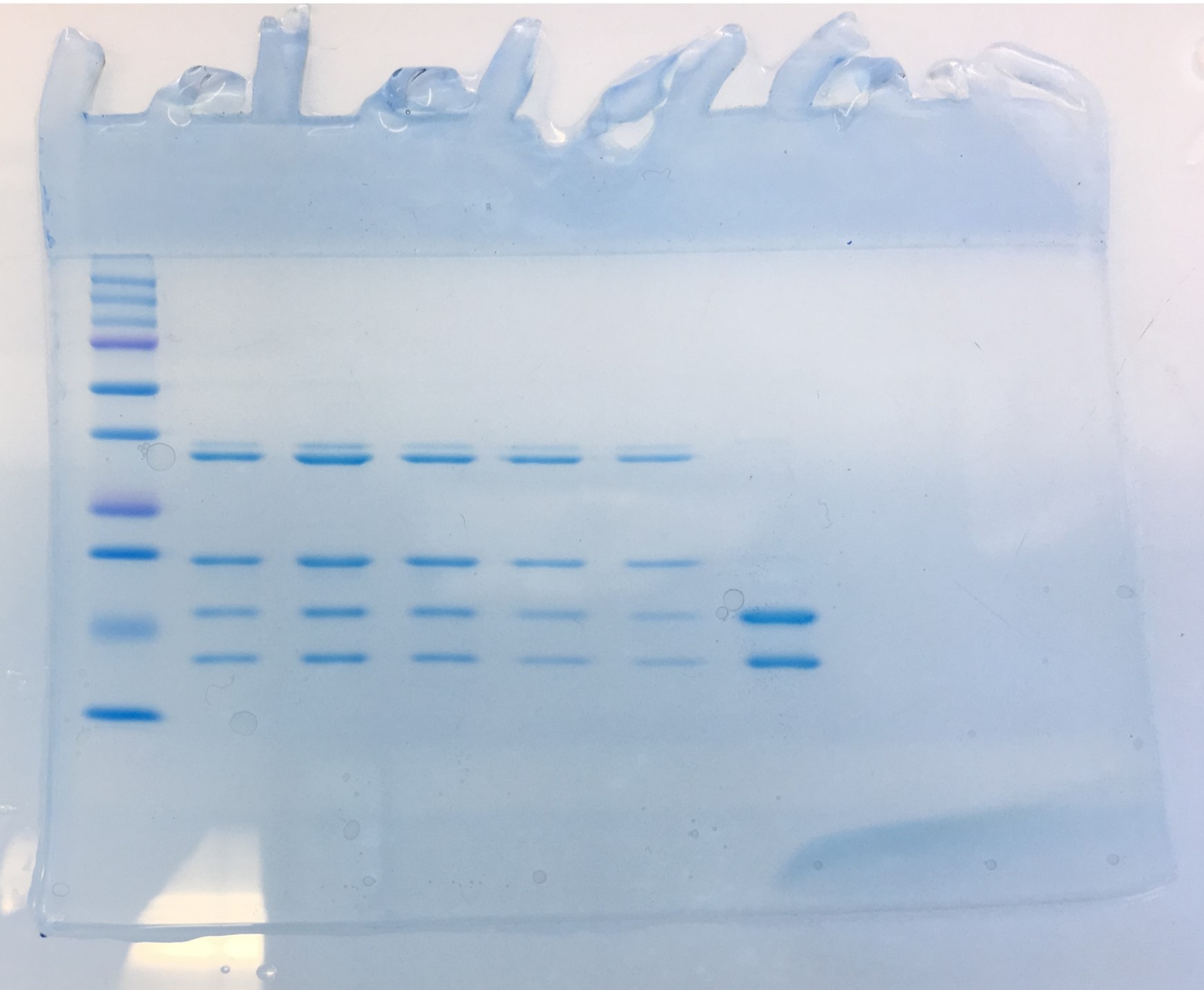

Supplement: Figure 8—source data 2. — This zipped folder contains the original files of the full raw unedited gel images for the size exclusion chromatography purification of each IC1-260 subcomplex. There is also a combined image with the uncropped gels with the relevant bands clearly labeled. [file elife-80217-fig8-data2.zip › Figure 8-gel1.jpg]

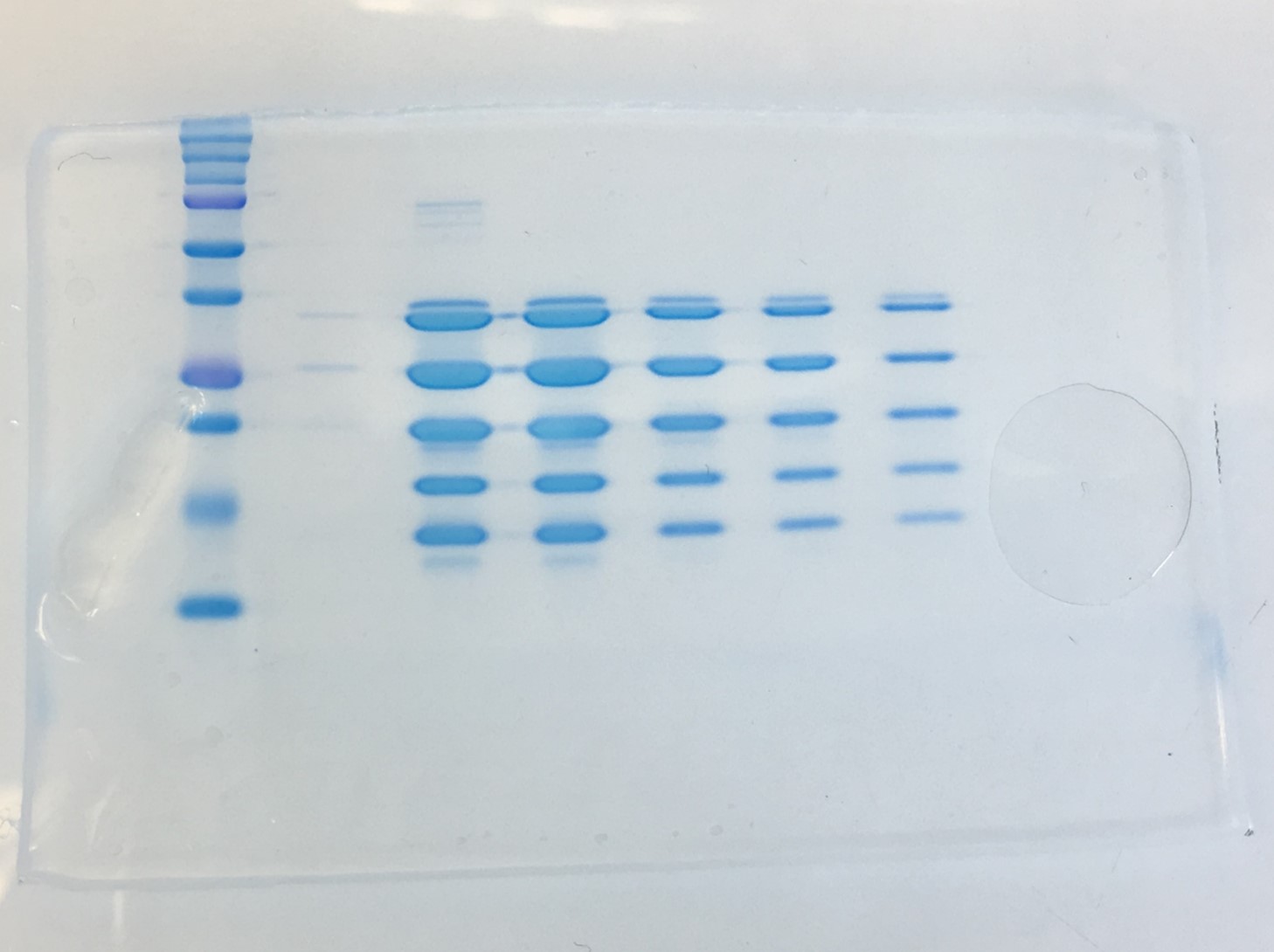

Supplement: Figure 8—source data 2. — This zipped folder contains the original files of the full raw unedited gel images for the size exclusion chromatography purification of each IC1-260 subcomplex. There is also a combined image with the uncropped gels with the relevant bands clearly labeled. [file elife-80217-fig8-data2.zip › Figure 8-gel2.jpg]

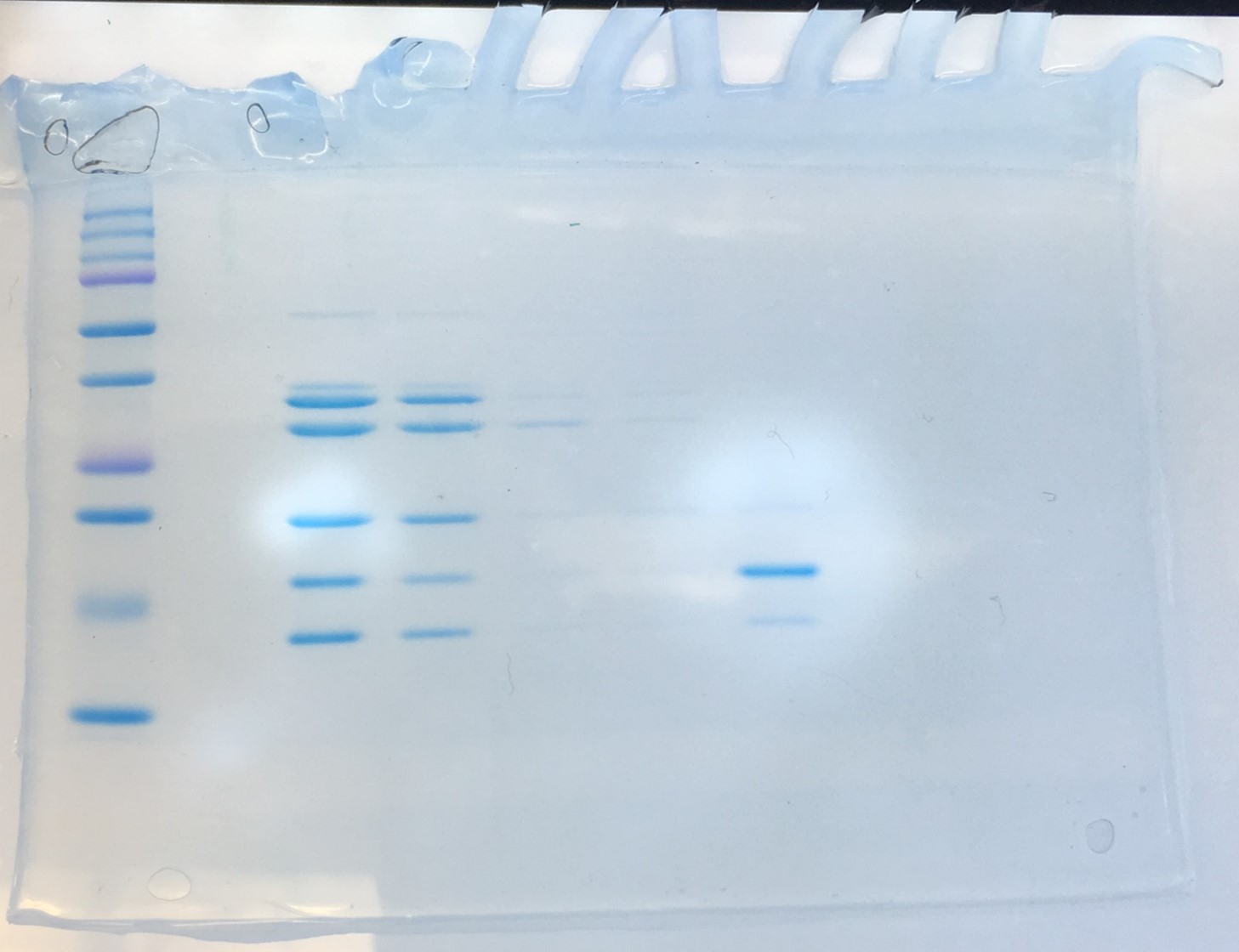

Supplement: Figure 8—source data 2. — This zipped folder contains the original files of the full raw unedited gel images for the size exclusion chromatography purification of each IC1-260 subcomplex. There is also a combined image with the uncropped gels with the relevant bands clearly labeled. [file elife-80217-fig8-data2.zip › Figure 8-gel3.jpg]

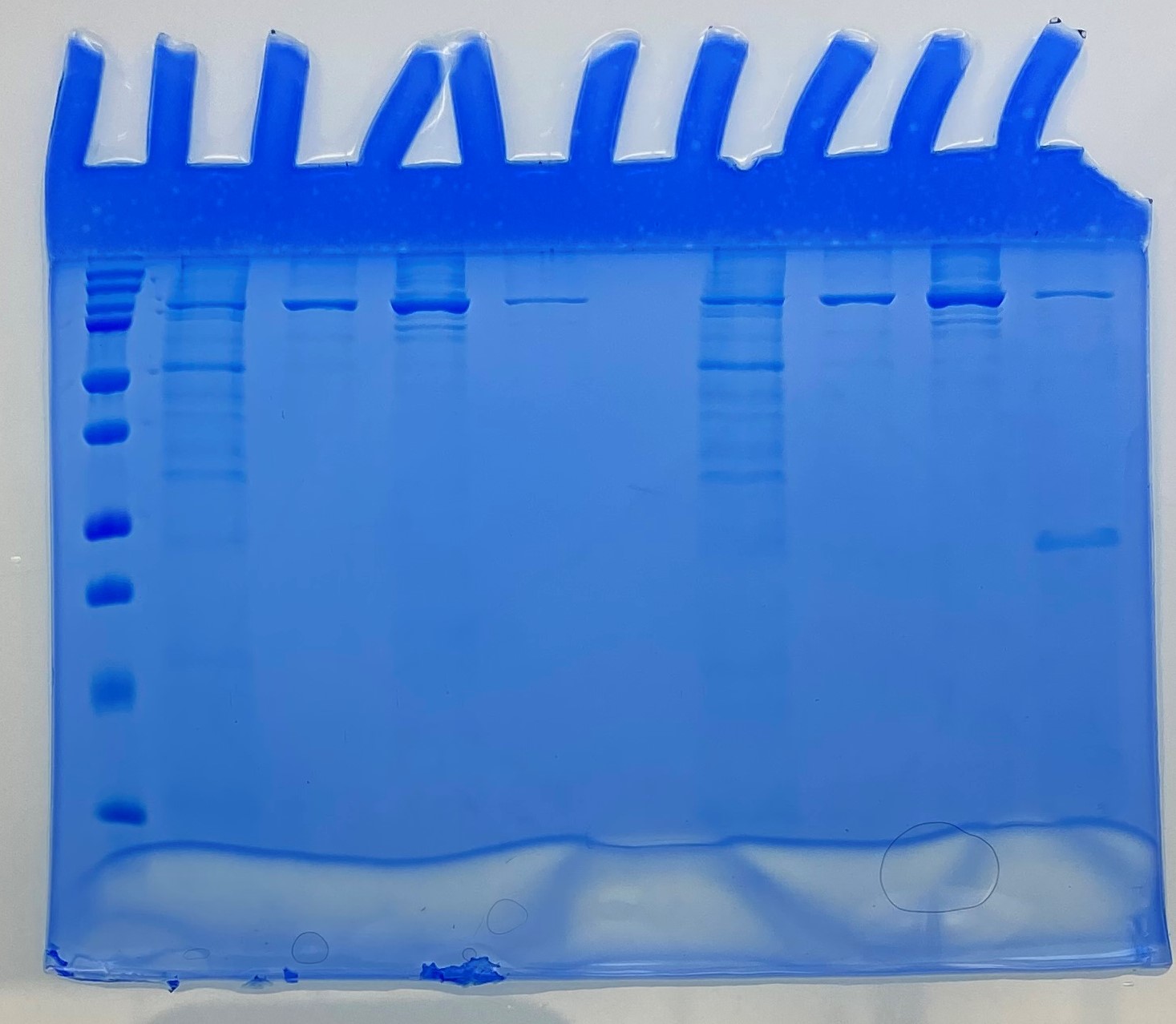

Supplement: Figure 9—source data 2. — This zipped folder contains the original file of the full raw unedited gel image for the IMAC fractions of ICFL. There is also an image with the uncropped gel with the relevant bands clearly labeled. Notice, two batches of ICFL were purified in tandem. [file elife-80217-fig9-data2.zip › Figure 9-source data 3.jpg]

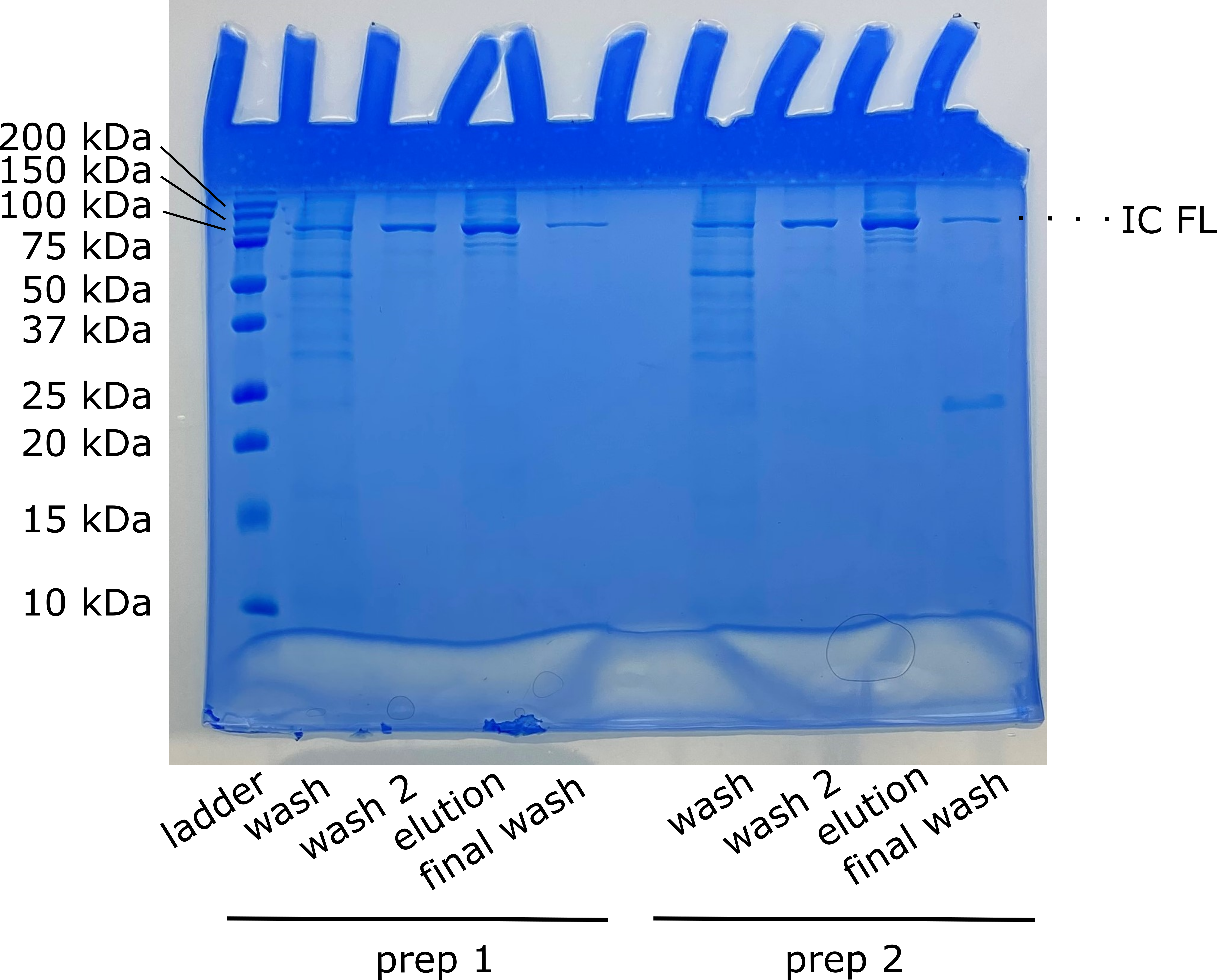

Supplement: Figure 9—source data 2. — This zipped folder contains the original file of the full raw unedited gel image for the IMAC fractions of ICFL. There is also an image with the uncropped gel with the relevant bands clearly labeled. Notice, two batches of ICFL were purified in tandem. [file elife-80217-fig9-data2.zip › Figure 9-source data 3.png]
