# Supplementary material for: Multivalency, autoinhibition, and protein disorder in the regulation of interactions of dynein intermediate chain with dynactin and the nuclear distribution protein
Source: eLife. 2022 Nov 23;11:e80217. doi: 10.7554/eLife.80217 (PMC9771362; doi:10.7554/eLife.80217)
Supplement: Supplementary file 1. — aFor the p150CC1B experiments, a two-site binding model was used as it provided much better fits when compared to a single site model. bFitting of this experiment was done with set N values due to the amount of extra noise in the data and thus the N values are reported without error. cThis experiment did not show evidence of binding and thus the data was not fit to a binding model. [file elife-80217-supp1.docx]

**Supplementary File 1: ITC results for IC interactions with p150_CC1B_ and NudE_CC_ at 25˚C**.

| **Binding Partner** | **CT IC Construct** | ***N*** | ***K*_d_  (µM)** | **Δ*G*˚ (kcal/mol)** | **Δ*H*˚ (kcal/mol)** | ***T*Δ*S*˚ (kcal/mol)** |
| --- | --- | --- | --- | --- | --- | --- |
| p150_CC1B_  (see note a) | IC_1-88_ | 0.49 ± 0.04 | 0.002 ± 0.0008 | -11.8 ± 0.2 | -4.2 ± 0.2 | 7.6 ± 0.3 |
|  |  | 0.50 ± 0.04 | 0.42 ± 0.13 | -8.7 ± 0.2 | 1.9 ± 0.2 | 10.6 ± 0.2 |
|  | IC_1-150_  (see note b) | 0.5 | 0.40 ± 0.21 | -8.7 ± 0.3 | 2.1 ± 0.3 | 10.8 ± 0.5 |
|  |  | 0.5 | 0.003 ± 0.002 | -11.6 ± 0.4 | -4.8 ± 0.2 | 6.8 ± 0.3 |
|  | IC_1-260_  (see note b) | 0.3 | 0.48 ± 0.05 | -8.6 ± 0.8 | 5.4 ± 0.5 | 14.1 ± 0.7 |
|  |  | 0.3 | 3.4 ± 0.5 | -7.5 ± 0.3 | -5.6 ± 0.5 | 1.8 ± 0.1 |
| NudE_CC_ | IC_1-88_ | 0.98 ± 0.01 | 0.34 ± 0.04 | -8.8 ± 0.2 | -5.1 ± 0.1 | 3.7 ± 0.2 |
|  | IC_1-150_ | 0.99 ± 0.01 | 0.34 ± 0.03 | -8.8 ± 0.1 | -8.8 ± 0.1 | 0.1 ± 0.1 |
|  | IC_1-260_ | N/A (See note c) | | | | |

^a^For the p150_CC1B_ experiments a two-site binding model was used as it provided much better fits when compared to a single site model.

^b^Fitting of this experiment was done with set *N* values due to the amount of extra noise in the data and thus the *N* values are reported without error.

^c^This experiment did not show evidence of binding and thus the data was not fit to a binding model.
